# Amelioration of acetic acid-induced ulcerative colitis in rats by cetirizine and loratadine via regulation of the PI3K/Akt/Nrf2 signalling pathway and pro-inflammatory cytokine release

**DOI:** 10.22038/IJBMS.2024.75889.16426

**Published:** 2024

**Authors:** Gihan F. Asaad, Rasha E. Mostafa

**Affiliations:** 1 Pharmacology Department, Medical Research and Clinical Studies Institute, National Research Centre, Cairo, Egypt

**Keywords:** Akt, Cetirizine, Loratadine, Nrf2, PI3K, Ulcerative colitis

## Abstract

**Objective(s):**

Ulcerative colitis is a chronic inflammatory bowel disease (IBD) that causes inflammation and ulcers in the rectum and the innermost layer of the large intestine. Our study aimed to elucidate the ameliorative effect of cetirizine (CTZ) and loratadine (LOR) against acetic acid-induced ulcerative colitis in rats via assessment of the PI3K/p-Akt/Nrf2 signaling pathway and proinflammatory cytokine release.

**Materials and Methods:**

Thirty-two rats were allocated into four groups (n=8). Group (I) was considered normal control. Acetic acid (AA) was injected intrarectally in groups (2-4). Group (2) was kept untreated. Group (3) was administered CTZ (20 mg/kg/day) for 7 days. Group (4) was administered LOR (10 mg/kg/day) for 7 days.

**Results:**

AA showed severe macroscopic colonic lesions associated with increased ulcer number, area, and severity with significantly elevated PI3K, p-Akt, Nrf2, TNF-α, and IL-6 in colorectal tissue as compared to the normal control group. All the aforementioned indicators were greatly improved by CTZ and LOR therapy.

**Conclusion:**

This is the first study to elucidate the ameliorative effect of CTZ and LOR against AA-induced UC in rats. CTZ and LOR treatment mitigates UC via amelioration of the PI3K/p-Akt/Nrf2 pathway and proinflammatory cytokine release.

## Introduction

Ulcerative colitis is a chronic inflammatory bowel disease (IBD) that causes inflammation and ulcers in the digestive tract. It affects the rectum and the innermost layer of the large intestine, generally known as the colon. Each person experiences ulcerative colitis differently, but common symptoms include diarrhea, passing blood in the stool, and stomach pain (1). Although the actual origin of ulcerative colitis is still unknown, scientists think that genes, aberrant immune responses, the environment, and the microbiome may all be involved (2). While there is now no recognized treatment for ulcerative colitis, there are several innovative therapies that can significantly lessen the disease’s signs and symptoms and result in long-lasting remission (3). According to a recent study, in 2023, the prevalence of ulcerative colitis was estimated to be 5 million cases around the world, and the incidence is increasing worldwide (4)the prevalence of ulcerative colitis was estimated to be 5 million cases around the world, and the incidence is increasing worldwide. Ulcerative colitis is thought to occur in people with a genetic predisposition following environmental exposures; gut epithelial barrier defects, the microbiota, and a dysregulated immune response are strongly implicated. Patients usually present with bloody diarrhoea, and the diagnosis is based on a combination of clinical, biological, endoscopic, and histological findings. The aim of medical management is, first, to induce a rapid clinical response and normalise biomarkers and, second, to maintain clinical remission and reach endoscopic normalisation to prevent long-term disability. Treatments for inducing remission include 5-aminosalicylic acid drugs and corticosteroids. Maintenance treatments include 5-aminosalicylic acid drugs, thiopurines, biologics (eg, anti-cytokines and anti-integrins. According to a different study, there were roughly 4.9 million cases of IBD worldwide in 2019, with China and the USA having the most cases (5). According to a more recent article, Northern Europe and North America have the highest incidence and prevalence of inflammatory bowel disease (6).

A well-known animal model for acute ulcerative colitis is acetic acid (AA)- induced ulcerative colitis in rats (7). In this paradigm, rats get intrarectal injections of acetic acid, which causes surface epithelium erosion, severe ulceration, mucosal atrophy, mucosal and submucosal hemorrhages, and inflammatory cell infiltration (8). The pathogenesis of ulcerative colitis is investigated using this model, and the effectiveness of prospective therapies for the condition is assessed (9).

5-aminosalicylic acid is typically used as the first step in the medical therapy of ulcerative colitis, followed by steroids and immunomodulators. To further intensify the treatment, calcineurin inhibitors (cyclosporine A and tacrolimus), infliximab, or surgery may be explored (10). Alternative methods of treating ulcers have been suggested, though. These include applying nicotine topically, consuming omega-3 fatty acids that have anti-inflammatory properties, and using biological agents like tumor necrosis factor alpha (TNF-α), interleukin (IL)-2 receptor, and anti-IL-12 and IL-6 antibodies (11). 

Cetirizine (CTZ), a second-generation antihistamine is a fast-acting, highly selective blocker of the peripheral histamine H1 receptor used to treat allergic rhinitis and urticaria. CTZ principally inhibits the H1 receptors on immunological cells, gastrointestinal tract cells, vascular endothelial cells, and respiratory smooth muscle cells (12). It has some ability to stabilize mast cells (13). CTZ does not pass the blood-brain barrier as much as first-generation antihistamines like diphenhydramine and doxylamine, avoiding the neurons of the central nervous system (14). CTZ is rapidly absorbed in the digestive system and significantly excreted via the kidneys (12). 

Loratadine (LOR), a long-acting, non-sedating antihistamine is used to treat allergies (15). It functions by inhibiting histamine, a chemical in the body that triggers allergic reactions (16). LOR preferentially inhibits H1-receptors, which are mostly found in immune cells, gastrointestinal tract cells, vascular endothelial cells, and respiratory smooth muscle cells (17). It binds to H1 histamine receptors on the surfaces of vascular, eosinophilic, and neutrophilic cells, as well as epithelial and endothelial cells. LOR has a weak affinity for CNS H1 receptors and does not enter the central nervous system very well. Due to these characteristics, CNS depressant effects such as drowsiness, sedation, and reduced psychomotor performance are absent (18).

It has been demonstrated that apart from the antihistaminic properties, CTZ was found to block the production of the cytokines IL-1β as well as down-regulate the expression of NF-κβ (19). LOR, an antihistamine used to treat allergies, has similarly been demonstrated to reduce inflammation by selectively targeting TAK1 and preventing the AP-1 signaling pathway’s activation and the subsequent generation of inflammatory cytokines (20). The information shows that both CTZ and LOR have anti-inflammatory properties in addition to blocking H1 receptors. One of the pathways implicated in acetic acid-induced ulcerative colitis is PI3k/p-Akt/Nrf2 (21). Akt phosphorylation can be used to indicate PI3K activity (22) where the activated Akt is released from the cell membrane and deposited in the cytoplasm or nucleus where it may continue to relay the signals and perform its biological functions. Nrf2 is a key transcription factor for regulating antioxidant proteins, which can boost antioxidant defense and reduce cell death. It is also one of the downstream substrates of the PI3K/AKT pathway (23). Therefore, this research aimed to evaluate the ameliorative effect of CTZ and loratadine LOR against AA-induced UC in rats via assessment of the PI3K/p-Akt/Nrf2 signaling pathway and proinflammatory release of the cytokines TNF-α and IL-6.

## Materials and Methods

***Animals***

Thirty-two male Wistar rats were purchased from the National Research Centre’s animal breeding unit (Dokki-Giza) and weighed (120-150 gr). Animals were housed in plastic cages with unlimited access to food and water. A 12-hour light/12-hour dark cycle was used to maintain the room temperature at 20±1 ^°^C. Following the guidelines of good hygiene, the cadavers of the animals used in the experiment were handled with care; direct contact with dead bodies were made immediately following termination, and they were submitted to the NRC Safety and Health Committee. Experiments were conducted following the Ethics and Animal Care Committee of the National Research Centre and following the recommendations of the National Institutes of Health Guide for Care and Use of Laboratory Animals (NIH Publications No. 8023, revised 1978).

***Chemical and kits***

Acetic acid (El-Nasr Pharmaceutical Co., Egypt). ELISA kits: Phosphatidylinositol-3 kinase (PI3K) (CUSABIO TECHNOLOGY LLC., Houston, TX 77054, USA; Cat. No.: CSB E08418r ), protein kinase B (p-Akt) (Bioassay Technology Laboratory, Shanghai, China; Cat. No.: E2452Ra), nuclear factor erythroid 2-related factor 2 (Nrf2) (Reddot Biotech. Inc., Houston, TX 77494, USA; Cat. No.: RD-NFE2L2-Ra) tumor necrosis factor-alpha (TNF-α) (BioLegend, Inc., San Diego, CA 92121, USA; Cat. No.: 438204), and Interleukin-6 (IL-6) (CLOUD-CLONE CORP., Katy, TX 77494, USA; Cat. No.: SEA079Ra). All other chemicals used were of the highest commercial grade available.

***Experimental design***

A) Induction of ulcerative colitis: single intrarectal injection of 2 ml acetic acid (3% w/v)(24) through a lubricated catheter under light sedation with ether for induction of ulcerative colitis (25). 

B) Treatments: Groups 3 and 4 were given drugs for 1 week after intrarectal injection with acetic acid (2 ml/rat; 3% (w/v). The doses of CTZ (20 mg/kg) and LOR (10 mg/kg) were selected based on earlier research (26, 27). 

32 rats in total were divided into the following four groups at random (n=8): Group 1: normal control group given normal saline; Group 2: acetic acid (AA) group (2 ml/rat, 3% w/v); Group 3: CTZ (20 mg/kg); and Group 4: LOR (10 mg/kg). Gastric lavage was used to provide medication orally. Twenty-four hours after the last treatment, rats were fasted and euthanized by cervical dislocation under midazolam 10 mg/kg anesthesia (28). 

***Macroscopical examination and ulcerative colitis scoring***

After opening the colon lengthwise, gently cleaning it underwater, and separating the colon (8 cm), it was macroscopically inspected for the number of ulcers, ulcer area, and ulcer severity (29). A magnifying lens was used to check for the development of gastric ulcers. Ulcer area (%) was calculated according to the following equation (Ut/Ca)*100, where; Ut= areas of all detected colon ulcers, Ca= area of the dissected colon. Ulcer scoring was made as previously described (30) as follows: 0; no damage, 1; blood at lumen, 2; pinpoint erosions, 3; one to five small erosions <2 mm, 4; more than five small erosions <2 mm, 5; One to three large erosions >2 mm, 6; more than three large erosions >2 mm.

***ELISA assessment of the PI3K, Akt, and Nrf2 levels in the homogenized colon tissue***

 A part of the colon was homogenized in 10% phosphate buffer saline (PBS). The aliquot was centrifuged at 1500×g at 4 ^°^C for 15 min and the supernatant was collected and stored at -80 ^°^C until used for the ELISA test. For assessment of phosphatidylinositol-3 kinase (PI3K), protein kinase B (Akt), nuclear factor erythroid 2-related factor 2 (Nrf2), tumor necrosis factor-alpha (TNF-α) and Interleukin-6 (IL-6) were assessed using ELISA rat kits according to manufacturers’ instructions. Antibodies specific for PI3K, p-Akt, Nrf2, IL6, and TNF-α had been pre-coated onto a microplate. Standards and samples are pipetted into the wells and any biomarker present is bound by the immobilized antibody. After removing any unbound substances, a biotin-conjugated antibody specific for PI3K, p-Akt, Nrf2, IL6, and TNF- α was added to the wells. After washing, avidin-conjugated Horseradish Peroxidase (HRP) is added to the wells. Following a wash to remove any unbound avidin-enzyme reagent, a substrate solution is added to the wells and color develops in proportion to the amount of PI3K, p-Akt, Nrf2, IL6, and TNF- α bound in the initial step. The color development is stopped and the intensity of the color is measured at a wavelength of 450 nm±10 nm.

***Statistical analysis***

Data statistical analysis was done using one-way analysis of variance (ANOVA) followed by Tukey’s *post hoc*. GraphPad (Graph Pad Software, Inc., San Diego, CA, USA) was employed for data analysis and graphical demonstrations. *P*>0.05 was considered significant. 

## Results

***Macroscopical examination***

**Figure 1:**
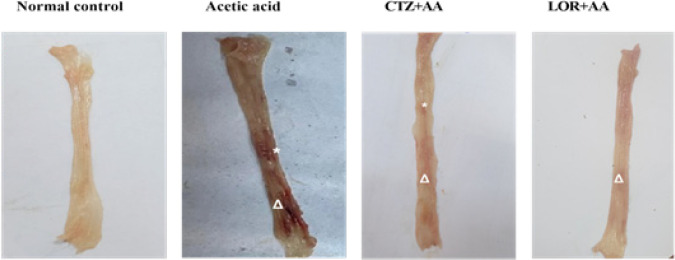
Macroscopical appearance of colon specimens (8 cm) showing normal intestinal mucosa in wister rat

Single intrarectal injection of AA (2 ml/rat, 3% w/v) caused severe damage in the intestinal mucosa which is demonstrated as a severe bloody lumen with many scattered pinpointed and longitudinal erosions extending along the length of the colon as compared to the normal controlled group. Treatment with both CTZ (20 mg/kg) and LOR (10 mg/kg) for 1 week following the induction of ulcerative colitis ameliorated the intestinal lesions as compared to the AA group. Data are depicted in Figure 1.

***Ulcerative colitis severity and scoring***

**Figure 2:**
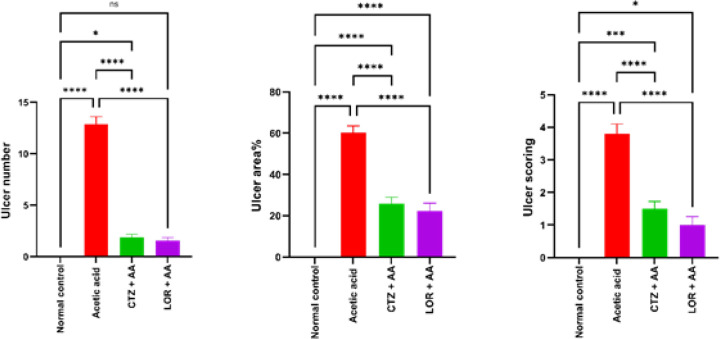
Effect of CTZ (20 mg/kg) and LOR (10 mg/kg) on ulcer number, severity, and score in wister rats with acetic acid-induced ulcerative colitis

A single administration of acetic acid (3% w/v) intrarectally resulted in a significant (*P*>0.0001) increase in ulcer number, ulcer area, and ulcer scoring (12.86±0.74, 60.4%±3.2 and 3.8±0.3, respectively) as compared to the normal control group which showed normal mucosa. Administration of CTZ (20 mg/kg) and LOR (10 mg/kg) for 7 days following induction of ulcerative colitis by acetic acid showed a significant *P*>0.0001 decrease in ulcer numbers (1.86±0.34 and 1.57±0.3), ulcer area% (25.9±3.1 and 22.32±3.76) and ulcer scoring (1.5±0.2 and 1±0.2), respectively as compared to the AA group. Data are depicted in Figure 2.

***Assessment of PI3K/p-Akt/Nrf2 pathway***

**Figure 3:**
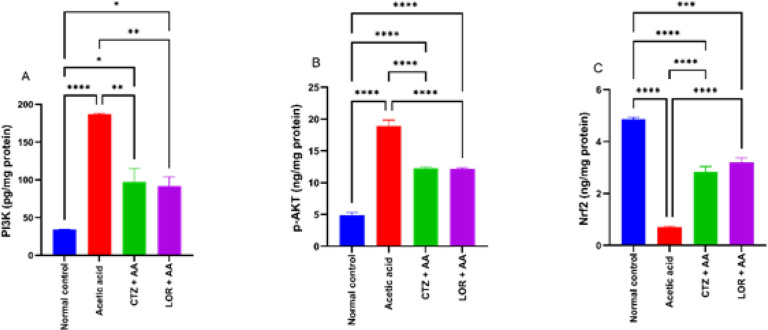
Effect of CTZ (20 mg/kg) and LOR (10 mg/kg) on PI3K/p-Akt/Nrf2 pathway in wister rats with acetic acid-induced ulcerative colitis

Intrarectal administration of acetic acid (3% w/v) in the AA group resulted in a significant *P*>0.0001 increase in PI3K and p-Akt (186.9±1.22 pg/mg protein & 18.93±0.91 ng/mg protein, respectively), and a significant (*P*>0.0001) decrease in Nrf2 (0.7±0.03 ng/mg protein) as compared to the normal control group (33.97±0.8 pg/mg protein, 4.93±0.4 ng/mg protein, and 4.86±0.07 ng/mg protein). Oral administration of CTZ (20 mg/kg) and LOR (10 mg/kg) for 7 days after induction of ulcerative colitis showed a significant (*P*>0.01) decrease in PI3K (97.27±3.24 and 91.45±3.55 pg/mg protein, respectively) as compared to the AA group showing 1.92 and 2.04 fold decrease respectively. In addition, CTZ (20 mg/kg) and LOR (10 mg/kg) showed significant (*P*>0.0001) decreases in p-Akt (12.26±0.24 and 12.17±0.18 ng/mg protein, respectively) as compared to the positive control group showing 1.54 and 1.56 fold decrease, respectively. On the other hand, CTZ (20 mg/kg) and LOR (10 mg/kg) showed a significant (*P*>0.0001) increase in Nrf2 (2.84±0.1 and 3.2±0.08 ng/mg protein, respectively) as compared to the AA group showing 4.06 and 4.57 fold increase, respectively. Our findings demonstrated that the anti-histaminic drugs CTZ and LOR may target PI3K/p-Akt/Nrf2 as an underlying mechanism for their ability to treat ulcerative colitis in rats produced by acetic acid. The data are shown in Figure 3.

***Assessment of proinflammatory cytokines***

**Figure 4:**
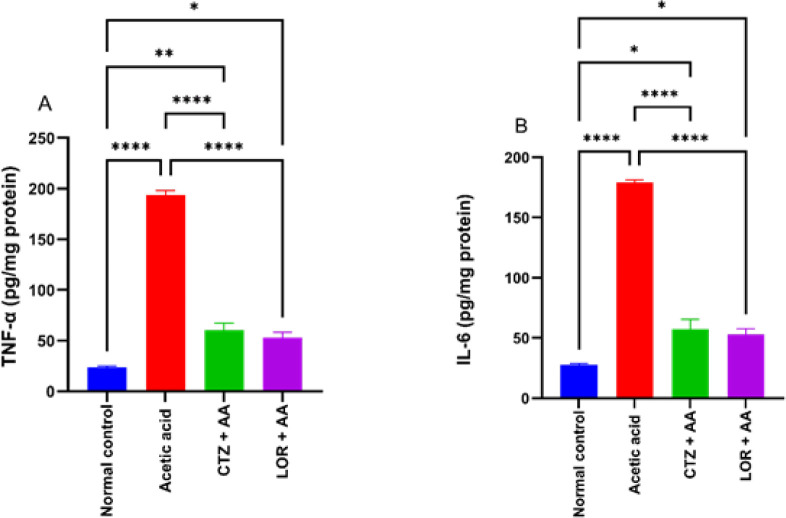
Effect of CTZ (20 mg/kg) and LOR (10 mg/kg) on the colonic proinflammatory cytokines; TNF-α, and IL-6 levels in wister rats with acetic acid-induced ulcerative colitis

A single administration of acetic acid (3% w/v) intrarectally in the AA group resulted in a significant (*P*>0.0001) increase in TNF-α and IL-6 (193.8±4.44 & 179.1±2.2 pg/mg protein, respectively) as compared to the normal control group (23.6±1.06 and 27.73±0.9 pg/mg protein, respectively). Oral administration of CTZ (20 mg/kg) and LOR (10 mg/kg) for 7 days after induction of ulcerative colitis, showed a significant *P*>0.05 decrease in TNF-α (60.39±1.97 and 53.02±0.76 pg/mg protein, respectively) as compared to AA group (3.21 and 3.66 fold decrease, respectively). In addition, CTZ (20 mg/kg) and LOR (10 mg/kg) showed significant *P*>0.0001 decreases in IL-6 (57.24±2.6 and 53.15±0.46 ng/mg protein, respectively) as compared to the AA group showing 3.13 and 3.37 fold decrease, respectively. Our data showed that CTZ and LOR can also exert their action via inhibition of the induced proinflammatory cytokine. Data are depicted in Figure 4.

## Discussion

The conditional relationship between histamine release and induction of ulcerative colitis via acetic acid has been studied extensively (31, 32), but their connection is not fully understood. It’s interesting to note that this served as our primary driving force in our quest to understand the potential therapeutic benefits and underlying mechanisms of the antihistamines CTZ and LOR concerning AA-induced ulcerative colitis and their ability to target PI3k/p-Akt/Nrf2 to be considered as a potential treatment for ulcerative colitis. In the current investigation, a single intrarectal injection of acetic acid caused severe mucosal damage, as evidenced by an increase in ulcer number, ulcer area, and ulcer severity. In accordance with our results, previous studies have reported that intrarectal administration of acetic acid has been shown to cause oxidative stress, inflammation, and apoptosis in the colon, which is marked by elevated neutrophil infiltration, extensive epithelial cell death, and ulceration (24, 33-35). We also reported that a single intrarectal administration of AA had activated the PI3K/p-Akt pathway via elevating the PI3K and p-Akt levels in colonic tissue, as well as a considerable down-regulation of the Nrf2 antioxidant defense system through reducing Nrf2 concentrations. Our results were in parallel with previous studies that reported that down-regulation of the PI3K/AKT signaling pathway was important in alleviating induced colitis via immunological modulation and anti-inflammatory effects (36, 37). It was also reported that activation of Nrf2 is an enormously sensitive sensor of oxidative stress and was found to be decreased in the ulcerative colitis group (21).

In the current study, intrarectal administration of AA leads to increased production of pro-inflammatory cytokines (IL-6 and TNF-α). In a previous study conducted by Ansari *et al*., 2021 (38), the authors reported that ulcerative colitis induced by AA is characterized by an immune system imbalance in the intestinal mucosa, including a dysregulated immune response and an imbalance in the release of pro-inflammatory cytokines (TNF-α and IL-6). Thus, effective control of these pro-inflammatory cytokines may represent a practical strategy for the management of UC.

In the current study, we discovered that oral administration of CTZ (20 mg/kg) and LOR (10 mg/kg) for seven days after acetic acid-induced ulcerative colitis significantly decreased the severity of the ulcer and the ulcer area, suggesting that both CTZ and LOR are capable of promoting tissue repair and maintaining mucosal integrity.

It has been noted that the p-Akt signal pathway, which regulates the creation and release of pro-inflammatory cytokines, is essential for the beginning and progression of UC (39). Accordingly, several studies have demonstrated that targeting the PI3K/p-Akt pathway is considered a potential therapeutic approach for rat ulcerative colitis (40-42). These findings show that inhibiting the PI3K/Akt pathway can enhance intestinal barrier integrity, restore the proper oxidant/antioxidant balance, lessen UC symptoms, and shield colitis mice from oxidative stress-induced damage. No prior research specifically examined the connection between PI3K/p-Akt and antihistamines in rats. In the present study, we discovered that CTZ and LOR significantly reduced the activation of the PI3K/p-Akt pathway brought about by acetic acid injection; consequently, these antihistamines demonstrated a promising potential therapeutic intervention point for induced ulcerative colitis and inflammatory bowel disease generally.

The search results indicate that in addition to their effects on H1 receptors, CTZ and LOR also have anti-inflammatory properties. Mast cells, basophils, lymphocytes, and other reservoirs produce histamine, a short peptide with inherent vasoactive properties, which interacts with histamine receptors to control a variety of cellular processes involved in allergic inflammation and immunological regulation. The H1-histamine receptor is the main target of suppressive therapy since it is most strongly linked to potentiation of proinflammatory immune cell activity and improved effector function (43, 44). The function of proinflammatory cytokines in ulcerative colitis (UC) in rats and the possibility of suppressing these cytokines to improve healing have been the subject of much research (38, 45). To find the best strategy for reducing these cytokines and encouraging healing in UC, more study is necessary. Our results were in parallel to these previous studies which showed a significant reduction in the proinflammatory cytokines TNF-α and IL-6 confirming the anti-inflammatory effect of CTZ and LOR. The combined action of cytokine pathways may explain the ability of anti-TNF-α therapies to both inhibit inflammation and promote mucosal healing. As a result, cytokine inhibition can aid in the treatment of ulcerative colitis.

Normal control, Acetic acid group with many scattered pinpointed (star) and longitudinal erosions (triangle), CTZ+AA group (20 mg/kg), and LOR+AA group (10 mg/kg) showing almost recovered mucosa represented by an obvious decrease in ulcer numbers and severity

AA: Acetic acid, CTZ: Cetirizine, LOR: Loratadine

Data is presented as mean±SEM (n=8). Statistical analysis was conducted by one-way analysis of variance (ANOVA) followed by Tukey-Kramer’s test for multiple comparisons (ns=non-significant, *P<0.05, ***P<0.001, ****P<0.0001)

CTZ: Cetirizine, LOR: Loratadine

Data is presented as mean±SEM (n=8). Statistical analysis was conducted by one-way analysis of variance (ANOVA) followed by Tukey-Kramer’s test for multiple comparisons (*P<0.05, **P<0.01, ****P<0.0001)

CTZ; Cetirizine, LOR: Loratadine, PI3k: Phosphatidylinositol-3 kinase, p-Akt: Protein kinase B, Nrf2: Nuclear factor erythroid 2-related factor 2

Data is presented as mean±SEM (n=8). Statistical analysis was conducted by one-way analysis of variance (ANOVA) followed by Tukey-Kramer’s test for multiple comparisons (*P<0.05, **P<0.01, ****P<0.0001)

CTZ; Cetirizine, LOR; Loratadine, TNF-α; Tumor necrosis factor-α, IL-6; Interleukin-6

## Conclusion

The therapeutic effects of the antihistamines CTZ and LOR against AA-induced UC in rats are being clarified in this study for the first time. CTZ and LOR treatment mitigates UC via regulation of the PI3K/p-Akt/Nrf2 signaling axis. Our data showed that CTZ and LOR can also exert their action by inhibiting the release of the cytokines TNF-α and IL-6. To fully understand the possible underlying mechanisms of CTZ and LOR in the field of treating ulcerative colitis, more research is required to scout for different pathways exerted by CTZ and LOR for alleviating induced ulcerative colitis.

## Authors’ Contributions

GF A and RE M equally contributed to the study’s conception, design, material preparation, data collection, and data analysis. The paper was written by GF A. The final manuscript was read and approved by GF A and RE M.

## Funding

The authors received no funds.

## Data availability

All data will be available upon request.

## Conflicts of Interest

The authors declare no conflicts of interest
